# Improvement in coronary heart disease risk factors during an intermittent fasting/calorie restriction regimen: Relationship to adipokine modulations

**DOI:** 10.1186/1743-7075-9-98

**Published:** 2012-10-31

**Authors:** Cynthia M Kroeger, Monica C Klempel, Surabhi Bhutani, John F Trepanowski, Christine C Tangney, Krista A Varady

**Affiliations:** 1 Department of Kinesiology and Nutrition, University of Illinois at Chicago, Chicago, IL, USA; 2 Department of Clinical Nutrition, Rush University, Chicago, IL, USA

**Keywords:** Intermittent fasting, Calorie restriction, Liquid diet, Body weight, Visceral fat, Cholesterol, Coronary heart disease, Obese women

## Abstract

**Background:**

The ability of an intermittent fasting (IF)-calorie restriction (CR) regimen (with or without liquid meals) to modulate adipokines in a way that is protective against coronary heart disease (CHD) has yet to be tested.

**Objective:**

Accordingly, we examined the effects of an IFCR diet on adipokine profile, body composition, and markers of CHD risk in obese women.

**Methods:**

Subjects (n = 54) were randomized to either the IFCR-liquid (IFCR-L) or IFCR-food based (IFCR-F) diet for 10 weeks.

**Results:**

Greater decreases in body weight and waist circumference were noted in the IFCR-L group (4 ± 1 kg; 6 ± 1 cm) versus the IFCR-F group (3 ± 1 kg; 4 ± 1 cm). Similar reductions (P < 0.0001) in fat mass were demonstrated in the IFCR-L (3 ± 1 kg) and IFCR-F group (2 ± 1 kg). Reductions in total and LDL cholesterol levels were greater (P = 0.04) in the IFCR-L (19 ± 10%; 20 ± 9%, respectively) versus the IFCR-F group (8 ± 3%; 7 ± 4%, respectively). LDL peak particle size increased (P < 0.01) in the IFCR-L group only. The proportion of small LDL particles decreased (P < 0.01) in both groups. Adipokines, such as leptin, interleukin-6 (IL-6), tumor necrosis factor-alpha (TNF-alpha), and insulin-like growth factor-1 (IGF-1) decreased (P < 0.05), in the IFCR-L group only.

**Conclusion:**

These findings suggest that IFCR with a liquid diet favorably modulates visceral fat and adipokines in a way that may confer protection against CHD.

## Introduction

Intermittent fasting (IF) is a novel weight loss regimen that has been steadily growing in popularity over the past decade [1]. This diet strategy generally involves severe restriction (75-90% of energy needs) on 1–2 days per week. Though clinical trial evidence is still limited [2,3], preliminary findings indicate that IF may be effective for weight loss and coronary heart disease (CHD) risk reduction. For instance, two recent trials of IF demonstrate decreases in body weight of 7-9% and reductions in LDL cholesterol of 10% after 20–24 weeks of treatment [2,3]. While these data are encouraging, this diet therapy is limited in that a long duration of time, i.e. 24 weeks, is required to experience modest reductions in weight. One possible way to boost the rate of weight loss would be to combine IF with daily calorie restriction (CR). In following this protocol, the individual would fast one day per week, and then undergo mild CR, i.e. 20% restriction of energy needs, on 6 days per week. The incorporation of portion-controlled liquid meals may also enhance weight loss as it helps individuals to stay within the confines of their prescribed energy goals [4,5]. The effect of IF combined with CR (with or without liquid meals) on body weight and CHD risk has yet to be tested.

Although the mechanisms remain unclear, the lipid-lowering actions of dietary restriction protocols may be mediated, in part, by modulations in adipokines, i.e. fat-cell derived hormones and cytokines [6]. Leptin is an adipokine that plays a role in CHD development by increasing platelet aggregation [7], and stimulating the proliferation and migration of endothelial cells [8]. Interleukin-6 (IL-6) and tumor necrosis factor-alpha (TNF-alpha) are pro-inflammatory mediators released by adipose tissue that are strong independent predictors of CHD [9]. C-reactive protein (CRP) is produced by adipose tissue in response to a rise in IL-6 [10]. CRP may play a role in atherogenesis by binding to oxidized LDL and promoting foam cell formation [11]. Isoprostanes are another group of compounds released by adipose tissue [12]. Isoprostanes act as markers of oxidative stress, and have been shown to accumulate in atherosclerotic lesions of carotid arteries derived from CHD patients [12]. Insulin-like growth hormone-1 (IGF-1), another adipose tissue-derived protein, may play a role in the development of CHD by stimulating the proliferation of vascular smooth muscle cells [13]. Circulating concentrations of these hormones are dictated by regional fat distribution [14]. Excess visceral adiposity, as determined by an increased waist circumference, is related to an increased incidence of dyslipidemia [14]. Viscerally obese women (defined as a waist circumference >88 cm) have higher circulating levels of each of the above-mentioned adipokines, relative to subcutaneously obese women [15]. The ability of IFCR to reduce visceral fat mass, and in turn, improve circulating adipokine profile, remains unknown.

Accordingly, the objective of the present study was to examine the effect of IFCR with a liquid-diet or food based diet on body weight and lipid risk factors for CHD in obese women, and to evaluate how changes in adipokines are related to these modulations in vascular disease risk.

## Methods

### Subjects

Obese women were recruited from the Chicago area by means of advertisements placed on and around the University of Illinois campus. Seventy-seven individuals responded to the advertisements, and 60 were deemed eligible to participate after the preliminary questionnaire, body mass index (BMI) and waist circumference assessment. Key inclusion criteria were as follows: female, age 35–65 y, BMI between 30 and 39.9 kg/m^2^, waist circumference >88 cm**,** weight stable for 3 months prior to the beginning of the study, i.e. <5 kg weight loss or gain, non-diabetic, no history of cardiovascular disease, no history of cancer, sedentary or lightly active for 3 months prior to the beginning of the study, i.e. <3 h/week of light-intensity exercise at 2.5–4.0 metabolic equivalents (METS), non-smoker, and not taking weight loss, lipid-lowering, or glucose-lowering medications. Perimenopausal women were excluded from the study, and postmenopausal women (defined as absence of menses for 2 y) were required to maintain their current hormone replacement therapy regimen for the duration of the study. The experimental protocol was approved by the Office for the Protection of Research Subjects at the University of Illinois, Chicago, and all volunteers gave written informed consent to participate in the trial.

### Diet interventions

Subjects were randomized by way of a stratified random sample, based on BMI and age, into either the IFCR-liquid diet (IFCR-L) group (n = 30) or IFCR-food based diet (IFCR-F) group (n = 30). A random number table was used to randomize the subjects from each strata into the intervention groups. The 10-week trial consisted of two dietary phases: 1) a 2-week baseline weight maintenance period, and 2) an 8-week weight loss period.

#### Baseline weight maintenance diet (Week 1–2)

**Figure 1:**
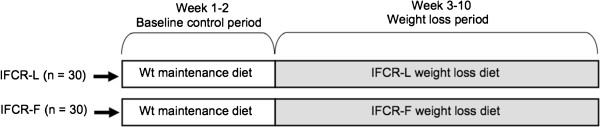
Experimental design.

Each subject participated in a 2-week baseline weight maintenance period before commencing the 8-week weight loss intervention (Figure 1). During this period, subjects were requested to continue eating their usual diet and to maintain a stable body weight.

#### Weight loss diets (Week 3–10)

After the baseline period, subjects partook in either the IFCR-L or IFCR-F intervention for 8 weeks (Figure 1). The Mifflin equation was used to measure energy requirements [16]. IFCR-L subjects (n = 30) consumed a calorie-restricted liquid diet for the first 6 days of the week, and then underwent a fast on the last day of the week (water consumption + 120 kcal of juice powder only, for 24 h). The liquid diet consisted of a liquid meal replacement for breakfast (240 kcal) and a liquid meal replacement for lunch (240 kcal). All liquid meal replacements were provided to the subjects in powder-form in premeasured envelopes (Isalean Shake, Isagenix LLC, Chandler, AZ). For the dinnertime meal, IFCR-L subjects consumed a 400–600 kcal meal. Food was not provided to the subjects for the dinner meal. Instead, subjects met with a Registered Dietician weekly to learn how to make healthy eating choices that are in compliance with the National Cholesterol Education Program Therapeutic Lifestyle Changes (TLC) diet (i.e. <35% of kcal as fat; 50-60% of kcal as carbohydrates; <200 mg/d of dietary cholesterol; and 20–30 g/d of fiber). In following this 7 d intervention, IFCR-L subjects were energy restricted by 30% of their baseline needs. IFCR-F subjects (n = 30) consumed a calorie-restricted food-based diet for the first 6 days of the week, and then underwent a fast on the last day of the week (water consumption + 120 kcal of juice powder only, for 24 h). IFCR-F subjects ate 3 meals per day in accordance with the TLC diet guidelines. Food was not provided to the subjects. Instead, subjects met with a Registered Dietician weekly to learn how to make healthy eating choices by implementing the TLC guidelines. Subjects were instructed to eat approximately 240 kcal for breakfast, 240 kcal for lunch, and 400–600 kcal for dinner. In following this 7 d intervention, IFCR-F subjects were energy restricted by 30% of their baseline needs.

### Analyses

#### Dietary intake and physical activity assessment

A multiple-pass, telephone-administered, 24-h recall was used to assess dietary intake. The recalls were performed at weeks 1, 3 and 10 by a trained Registered Dietician. Dietary intake data were analyzed using Nutrition Data System (NDS) software (version 2012; University of Minnesota, Minneapolis, MN). Furthermore, IFCR-L subjects were provided with a checklist each day to monitor: 1) adherence to the liquid meal protocol, and 2) adherence to the fast day regimen. IFCR-F subjects were also given a checklist to monitor their adherence to the fast day regimen. Alterations in energy expenditure associated with changes in physical activity were measured by the use of a pattern recognition monitor (Sense Wear Mini (SWM), Bodymedia, Pittsburgh, PA). Subjects wore the lightweight monitor on their upper arm for 7 d at week 3 and 10 of the trial. The data was analyzed using Bodymedia Software V.7.0, algorithm V.2.2.4 (Bodymedia, Pittsburgh, PA).

#### Hunger, satisfaction, and fullness assessment

Subjects completed a validated visual analog scale (VAS) on each fast day approximately 5 min before going to bed (reported bedtime ranged from 8.00 pm to 1.20 am). In brief, the VAS consisted of 100-mm lines, and subjects were asked to make a vertical mark across the line corresponding to their feelings from 0 (not at all) to 100 (extremely) for hunger, satisfaction with diet, or fullness. The VAS was collected at the weigh-in each week and reviewed for completeness. Quantification was performed by measuring the distance from the left end of the line to the vertical mark.

#### Body weight and body composition assessment

Body weight measurements were taken to the nearest 0.5 kg at the beginning of every week in light clothing and without shoes using a balance beam scale (HealthOMeter; Sunbeam Products, Boca Raton, FL). Height was assessed using a wall-mounted stadiometer to the nearest 0.1 cm. BMI was assessed as kg/m^2^. Fat mass and fat free mass were assessed by dual energy X-ray absorptiometry (DXA) at weeks 1, 3 and 10 (QDR 4500 W, Hologic Inc. Arlington, MA). Waist circumference was measured by a flexible tape to the nearest 0.1 cm, midway between the lower costal margin and super iliac crest during a period of expiration.

#### Plasma lipids and adipokine assessment

Fasting blood samples were collected between 6.00 am and 9.00 am at weeks 1, 3 and 10 after a 12-h fast. Blood was centrifuged for 10 min at 520 × g at 4°C to separate plasma from red blood cells and was stored at −80°C until analyzed. Plasma total cholesterol, direct LDL cholesterol, HDL cholesterol, and triglyceride concentrations were measured in duplicate by enzymatic kits (Biovision Inc, Mountainview, CA). LDL particle size was measured by linear polyacrylamide gel electrophoresis (Quantimetrix Lipoprint System, Redondo Beach, CA), as previously described [17]. Leptin, IL-6, TNF-alpha, CRP, 8-isoprostane, and IGF-1 were assessed in duplicate at week 1, 3, and 10 by ELISA (R&D Systems, Minneapolis, MN).

#### Statistics

Results are presented as mean ± SEM. Sample size was calculated as n = 30 subjects per group, assuming a 5% decrease in body weight in the IFCR-L group, with a power of 80% and an α risk of 5%. An independent samples *t*-test was used to test baseline differences between groups. Repeated-measures ANOVA was performed, taking time as the within-subject factor and diet as the between-subject factor, to assess differences between groups over the course of the study. Post-hoc analyses were performed using the Tukey test. Differences were considered significant at P < 0.05. All data was analyzed using SPSS software (version 20.0, SPSS Inc, Chicago, IL).

## Results

### Subject dropout and baseline characteristics

Sixty subjects (IFCR-L n = 30, IFCR-F n = 30) commenced the study. Two subjects dropped out of the IFCR-L group due to scheduling conflicts (n = 1) and problems adhering to the diet (n = 1). Four subjects dropped out of the IFCR-F group because of scheduling conflicts (n = 2) and inability to adhere to the diet protocol (n = 2). Thus, a total of 28 and 26 subjects completed the IFCR-L and IFCR-F interventions, respectively. There were no differences between groups for age, ethnicity, or BMI (Table 1).

**Table 1:** **Subject characteristics at baseline**^**1**^

**Characteristic**	**IFCR-L**	**IFCR-F**
n	28	26
Age (y)	47 ± 2	48 ± 2
Ethnicity		
African American	16	18
Asian	3	2
Caucasian	4	2
Hispanic	5	4
Body weight (kg)	95 ± 3	94 ± 3
Height (cm)	165 ± 2	164 ± 2
Body mass index (kg/m^2^)	35 ± 1	35 ± 1

^1^ Values reported as mean ± SEM. IFCR-L: Intermittent fasting calorie restriction-liquid diet; IFCR-F: Intermittent fasting calorie restriction-food based diet. No differences between groups for any parameter (Independent samples *t*-test).

### Dietary intake and physical activity

Diet and physical activity data are displayed in Table 2. Adherence to the fast day protocol was similar between groups (P = 0.91) (IFCR-l: 96 ± 4% compliance; IFCR-F: 98 ± 3% compliance). Compliance with the liquid meal protocol was 92 ± 3% in the IFCR-L group over the course of the 8 weeks. Energy intake decreased (P < 0.05) in both the IFCR-L and IFCR-F groups between week 3 and 10. There were no changes in fat, protein, carbohydrate, cholesterol, or fiber intake from the beginning to the end of the study in either group. Activity expenditure and steps/d remained stable over the course of the trial in both intervention groups. Hours of sleep per night also did not change during the 8-week weight loss period in either the IFCR-L or IFCR-F group.

**Table 2:** **Dietary intake and physical activity during the weight loss period**^1^

		**IFCR-L**			**IFCR-F**	
**Diet variables**	**Week 3**	**Week 10**	**Change**^**2**^	**Week 3**	**Week 10**	**Change**^**2**^
Energy (kcal)	1708 ± 135	1255 ± 102 ^3^	−453 ± 210	1694 ± 180	1444 ± 132 ^3^	−250 ± 146
Total fat (g)	54 ± 6	51 ± 11	−3 ± 14	62 ± 9	47 ± 5	−15 ± 11
Saturated fat (g)	20 ± 2	18 ± 3	−2 ± 4	22 ± 4	18 ± 2	−4 ± 4
Monounsaturated fat (g)	22 ± 2	17 ± 3	−5 ± 5	26 ± 4	17 ± 1	−9 ± 4
Polyunsaturated fat (g)	12 ± 2	16 ± 5	4 ± 6	14 ± 2	12 ± 2	−2 ± 3
Protein (g)	75 ± 5	84 ± 12	9 ± 14	67 ± 6	65 ± 9	−2 ± 4
Carbohydrates (g)	226 ± 20	215 ± 49	−11 ± 60	210 ± 23	174 ± 20	−36 ± 20
Cholesterol (mg)	215 ± 27	196 ± 36	−19 ± 51	224 ± 43	169 ± 28	−55 ± 53
Fiber	17 ± 1	23 ± 7	6 ± 8	20 ± 2	16 ± 2	−4 ± 2
Physical activity variables	Week 3	Week 10	Change ^2^	Week 3	Week 10	Change ^2^
Activity expenditure (kcal/d)	249 ± 28	283 ± 27	34 ± 76	246 ± 37	258 ± 43	12 ± 25
Steps (steps/d)	6975 ± 526	7375 ± 426	400 ± 261	5876 ± 621	6405 ± 599	529 ± 610
Sleep (h/d)	6.4 ± 0.4	6.0 ± 0.2	−0.4 ± 1.0	6.2 ± 0.5	5.5 ± 0.4	0.7 ± 0.5

^1^ Values reported as mean ± SEM. IFCR-L: IFCR-L: Intermittent fasting calorie restriction-liquid diet (n = 28); IFCR-F: Intermittent fasting calorie restriction-food based diet (n = 26).

^2^ Change expressed as the difference between week 3 and week 10 absolute values.

^3^ Week 3 values significantly (P < 0.05) different from week 10 values within group (Repeated-measures ANOVA).

No significant difference between groups for any nutrient except energy.

### Hunger, satisfaction, and fullness assessment

Hunger scores were low, and did not differ over the course of the trial in the IFCR-L (week 3: 27 ± 8 mm, week 10: 28 ± 7 mm) or IFCR-F group (week 3: 46 ± 7 mm, week 10: 39 ± 7 mm). Satisfaction with the diets remained elevated from the beginning to the end of the study in the IFCR-L (week 3: 72 ± 7 mm, week 10: 78 ± 6 mm) and IFCR-F group (week 3: 55 ± 9 mm, week 10: 66 ± 6 mm). Fullness scores were moderate and stable during the trial in the IFCR-L (week 3: 66 ± 7 mm, week 10: 82 ± 6 mm) or IFCR-F group (week 3: 58 ± 7 mm, week 10: 64 ± 6 mm). No differences were noted between groups for hunger, satisfaction or fullness at either time point.

### Body weight and body composition

Changes in body weight and body composition are reported in Table 3. Body weight remained stable in both the IFCR-L group (week 1: 95 ± 3, week 3: 95 ± 3 kg) and IFCR-F group (week 1: 94 ± 3, week 3: 94 ± 3 kg) during the weight maintenance period. Body weight decreased to a greater extent (P < 0.05) in the IFCR-L group (4 ± 1 kg) versus the IFCR-F group (3 ± 1 kg) during the weight loss period. Similar decreases (P < 0.0001) in fat mass were observed in the IFCR-L (3 ± 1 kg) and IFCR-F (2 ± 1 kg) groups after 8 weeks of treatment. Fat free mass remained unchanged throughout the course of the trial in both groups. Greater decreases (P < 0.05) in waist circumference were demonstrated in the IFCR-L (6 ± 1 cm) when compared to the IFCR-F group (4 ± 1 cm). BMI decreased (P < 0.0001) by 1 ± 1 and 1 ± 1 kg/m^2^, respectively, in the IFCR-L and IFCR-F groups.

**Table 3:** **Body weight and body composition changes during the weight loss period**^**1**^

		**IFCR-L**			**IFCR-F**	
	**Week 3**	**Week 10**	**Change**^**2**^	**Week 3**	**Week 10**	**Change**^**2**^
Body weight (kg)	95 ± 3	91 ± 3 ^3^	−4 ± 1 ^4^	94 ± 2	91 ± 2 ^3^	−3 ± 1
Fat mass (kg)	45 ± 1	42 ± 1 ^3^	−3 ± 1	45 ± 1	43 ± 1 ^3^	−2 ± 1
Fat free mass (kg)	50 ± 1	49 ± 2	−1 ± 1	49 ± 1	48 ± 1	−1 ± 1
Waist circumference (cm)	103 ± 1	97 ± 1 ^3^	−6 ± 1 ^4^	102 ± 3	98 ± 3 ^3^	−4 ± 1
Body mass index (kg/m^2^)	35 ± 1	34 ± 1 ^3^	−1 ± 1	35 ± 1	34 ± 1 ^3^	−1 ± 1

^1^ Values reported as mean ± SEM. IFCR-L: IFCR-L: Intermittent fasting calorie restriction-liquid diet (n = 28); IFCR-F: Intermittent fasting calorie restriction-food based diet (n = 26).

^2^ Change expressed as the difference between week 3 and week 10 absolute values.

^3^ Week 3 values significantly (P < 0.05) different from week 10 values within group (Repeated-measures ANOVA).

^4^ Absolute change significantly different (P < 0.05) between the IFCR-L and IFCR-F group (Repeated-measures ANOVA).

### Plasma lipids and adipokines

**Figure 2:**
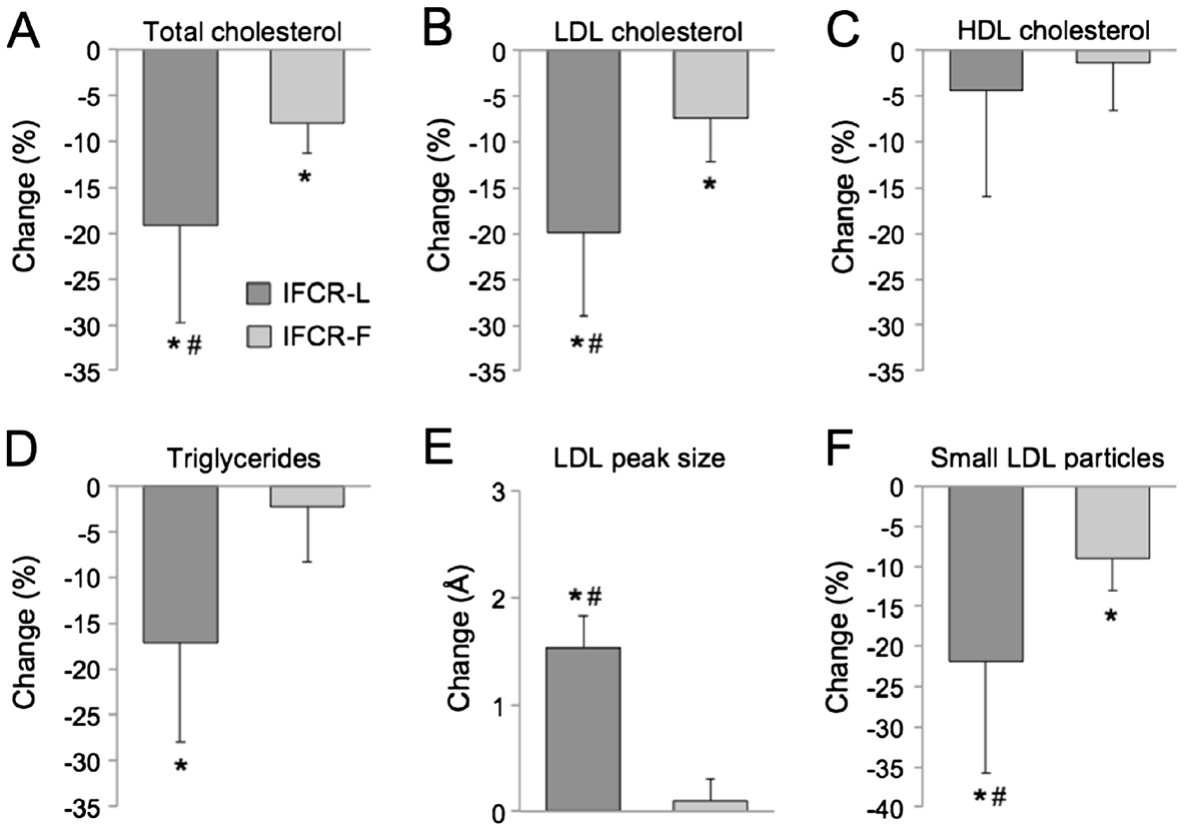
**Changes in lipid indicators of coronary heart disease risk during the weight loss period.** Values reported as mean ±SEM change between week 3 and 10. IFCR-L: Intermittent fasting calorie restriction-liquid diet (n = 28); IFCR-F. Intermittent fasting calorie restriction-food based diet (n = 26). **A.** Total cholesterol. **B.** LDL cholesterol. **C.** HDL cholesterol. **D.** Triglycerides. **E.** LDL peak particle size. **F.** Proportion of small LDL particles. *Week 3 values significantly (P < 0.05) different from week 10 values within E group (Repeated-measures ANOVA). # Significantly different (P < 0.05) between the IFCR-L and IFCR-F group (Repeated-measures ANOVA).

Changes in plasma lipid concentrations and LDL particle size are displayed in Figure 2. Total and LDL cholesterol concentrations were reduced (P < 0.05) to a greater extent in the IFCR-L group (19 ± 10%; 20 ± 9%, respectively) compared to the IFCR-F group (8 ± 3%; 7 ± 4%, respectively). HDL cholesterol was not changed by either diet. Triglycerides decreased (P < 0.0001) in the IFCR-L group only (17 ± 9%). LDL peak particle size increased (P < 0.01) in the IFCR-L group only, while the proportion of small particles was reduced (P < 0.05) in the both the IFCR-L and IFCR-F groups. Changes in adipokines are reported in Table 4. Leptin decreased (P < 0.05) to a similar extent in the IFCR-L (−9 ± 2 ng/ml) and the IFCR-F group (−8 ± 2 ng/ml). IL-6, TNF-alpha, and IGF-1 concentrations were reduced (P < 0.05) in the IFCR-L group only. Significant associations between plasma lipids and adipose tissue parameters were noted in the IFCR-L group only. For instance, decreases in LDL cholesterol were related to reductions in waist circumference (r = 0.26, P = 0.04), and leptin (r = 0.37, P = 0.04). Reductions in triglycerides were also related to decreased leptin (r = 0.29, P = 0.04) and TNF-alpha (r = 0.33, P = 0.03). Furthermore, decreased waist circumference was related to lower circulating leptin levels (r = 0.45, P = 0.03).

**Table 4:** **Changes in adipokines during the weight loss period**^**1**^

		**IFCR-L**			**IFCR-F**	
	**Week 3**	**Week 10**	**Change**^**2**^	**Week 3**	**Week 10**	**Change**^**2**^
Leptin (ng/ml)	36.5 ± 2.5	27.1 ± 3.1 ^3^	−9.4 ± 1.6	37.6 ± 2.5	29.4 ± 2.4 ^3^	−8.2 ± 1.7
IL-6 (pg/ml)	3.1 ± 0.3	2.5 ± 0.3 ^3^	−0.6 ± 0.3	3.1 ± 0.4	3.3 ± 0.4	0.2 ± 0.4
TNF-alpha (pg/ml)	1.6 ± 0.3	1.2 ± 0.3 ^3^	−0.4 ± 0.1	1.2 ± 0.3	1.1 ± 0.2	−0.1 ± 0.1
C-Reactive protein (mg/dl)	0.4 ± 0.1	0.4 ± 0.1	0 ± 0.1	0.6 ± 0.2	0.4 ± 0.1	−0.2 ± 0.2
8-isoprostane (pmol/mg)	1.8 ± 0.1	1.9 ± 0.1	0.1 ± 0.1	1.8 ± 0.1	1.9 ± 0.1	0.1 ± 0.1
IGF-1 (ng/ml)	67.7 ± 3.8	60.8 ± 4.2 ^3^	−6.9 ± 2.3	69.6 ± 3.8	67.6 ± 5.4	−2.0 ± 4.9

^1^ Values reported as mean ± SEM. IFCR-L: Intermittent fasting calorie restriction-liquid diet (n = 28); IFCR-F: Intermittent fasting calorie restriction-food based diet (n = 26). IL-6: Interleukin-6, TNF-alpha: Tumor necrosis factor-alpha, IGF-1: Insulin-like growth factor-1.

^2^ Change expressed as the difference between week 3 and week 10 absolute values.

^3^ Week 3 values significantly (P < 0.05) different from week 10 values within group (Repeated-measures ANOVA).

## Discussion

This study is the first to show that IFCR with liquid meals can produce potent decreases in CHD risk, and that these effects are mediated in part by improvements in adipokines. More specifically, we show here that IFCR-L is an effective diet therapy to modulate lipid indicators of CHD risk, i.e. reduce LDL cholesterol, triglycerides, and the proportion of small LDL particles, while increasing LDL peak particle size. Our findings also demonstrate that these favorable changes in lipids were related to reduced waist circumference (visceral fat mass) and reductions in pro-atherogenic adipokines, such as leptin and TNF-alpha.

Although both of the interventions produced favorable changes in lipids, superior modulations were shown in the IFCR-L group when compared to the IFCR-F group. The reductions in plasma lipids by IFCR-L (LDL-cholesterol: 19%, triglycerides: 20%) are similar to what has been reported in previous trials of IF [2,3]. For instance, in a trial by Williams et al. [3], obese subjects consumed a very-low calorie diet (VLCD; <500 kcal/d) 1 day per week, and ate ad libitum every other day of the week. After 20 weeks of treatment, LDL cholesterol and triglyceride concentrations decreased by 10% and 52%, respectively [3]. In the trial by Harvie et al. [2], obese women underwent 2 days of VLCD (600 kcal/d) and ate ad libitum on every other day of the week, for 24 weeks. Post-treatment LDL cholesterol and triglyceride levels were reduced by 10% and 17% from baseline [2]. The mechanism by which IFCR modulates circulating lipid concentrations is not clear. Nonetheless, recent evidence from CR studies indicate that the oxidation of circulating free fatty acids (FFA) is increased during periods of weight loss, while FFA synthesis is decreased [18]. Lower availability of precursor FFA results in a reduction in hepatic synthesis and secretion of very-low density lipoprotein (VLDL) into plasma. Lower secretion of VLDL contributes to reduced plasma concentrations of LDL, since VLDL is quickly converted to LDL in the circulation [19]. Although this mechanism has only been demonstrated for CR, it possible that IFCR may alter circulating lipids in a similar fashion.

The greater improvement in lipid profile by IFCR-L is most likely due to the more pronounced reductions in body weight and visceral fat mass observed. After 8 weeks of treatment, body weight and waist circumference, an indirect indicator of visceral fat, decreased to a greater extent in the IFCR-L (4 kg and 6 cm, respectively) when compared to the IFCR-F group (3 kg and 4 cm, respectively). The greater weight loss by the IFCR-L group is not surprising as these individuals had a larger daily calorie deficit relative to the IFCR-F group (453 kcal/d, 250 kcal/d, respectively). These decreases in waist circumference in the IFCR-L group were related to reductions in LDL cholesterol concentrations. An accumulation of adipose tissue in visceral depots may contribute to the development of dyslipidemia in several ways. For instance, lipolysis of fat tissue in visceral adipocytes is higher than that of subcutaneous adipocytes. This can lead to an augmented efflux of FFA from visceral depots [20]. These FFAs released from visceral fat are then collected by the portal vein and reach the liver at much higher concentrations than they do the systemic circulation [20]. In the liver, these FFA trigger the augmented production of triglycerides, and the elevated secretion of VLDL [20]. The increased secretion of VLDL then results in higher plasma levels of LDL [20]. In view of this, it is conceivable that the decrease in visceral fat by IFCR played a role in the reduced LDL cholesterol concentrations shown here.

Decreases in pro-atherogenic adipokines, such as leptin (26%), IL-6 (19%), TNF-alpha (25%), and IGF-1 (10%) were observed by the IFCR-L group. No changes in adipokines were noted in the IFCR-F group, however, which is most likely due to insufficient weight loss to achieve changes in these parameters [21]. The decreases in leptin noted here are similar to those observed by Harvie et al. [2]. After 24 weeks of IF, obese women experienced potent reductions in leptin of 40% [2]. The greater reductions in leptin in this previous trial are most likely due to the greater weight loss achieved with the longer trial duration [22]. In the IFCR-L group, lower plasma leptin was related to decreased triglyceride levels. In vitro studies demonstrate that leptin is a potent stimulator of lipolysis and fatty acid oxidation in adipocytes and other cell types [23]. Accordingly, leptin is also a regulator of circulating triacylglycerol concentrations [23]. Reduced leptin levels were also related to lower LDL cholesterol levels post-treatment, though the mechanism by which leptin may be involved in reducing LDL cholesterol concentrations is not clear. We also observed a positive association between visceral fat mass and leptin levels. Thus, it is possible that the decrease in visceral fat by the IFCR-L intervention stimulated a reduction in leptin, which in turn contributed to the lipid profile improvements demonstrated here. Reductions in TNF-alpha were also correlated to decreases in plasma triglycerides. Evidence in rodent models indicates that TNF-alpha induces hyperlipidemia by increasing hepatic triglyceride production [24]. Thus, a reduction in circulating TNF-alpha by IFCR-L may be related to the reductions in triglycerides observed.

This study is limited in that it did not carefully control for food intake by providing food-based meals to the intervention groups, i.e. dinner meal for the IFCR-L group, and 3 meals/d for the IFCR-F group. Providing all the meals during the study would allow for a more precise assessment of dietary adherence. Another disadvantage is that only female subjects were employed, and as such, the applicability of these findings to males remains uncertain.

Taken together, our results suggest that IFCR with liquid meals is an effective diet therapy to reduce body weight, visceral fat mass, and lipid indicators of CHD risk. Our findings also demonstrate that the beneficial modulations in vascular disease risk by IFCR may be mediated, in part, by reductions in visceral fat mass and pro-atherogenic adipokines. This study is an important first step to understanding the underlying mechanisms that mediate the cardio-protective effects of this novel diet regimen.

## Competing interests

KAV has a consulting relationship with the sponsor of the research, lsagenix, LLC.

## Authors’ contributions

CMK and MCK conducted the clinical trial, analyzed the data, and assisted with the preparation of the manuscript. SB and JFT performed the laboratory analyses, and assisted with data analyses. CCT performed the analysis of dietary intake data. KAV designed the experiment and wrote the manuscript. All authors read and approved the final manuscript.

## Funding source

This study was funded by Isagenix LLC., Chandler, AZ.
